# Impact of Sacubitril/Valsartan on Circulating microRNA in Patients with Heart Failure

**DOI:** 10.3390/biomedicines11041037

**Published:** 2023-03-28

**Authors:** Maura Brioschi, Yuri D’Alessandra, Massimo Mapelli, Irene Mattavelli, Elisabetta Salvioni, Sonia Eligini, Alice Mallia, Veronica Ricci, Erica Gianazza, Stefania Ghilardi, Piergiuseppe Agostoni, Cristina Banfi

**Affiliations:** 1 Centro Cardiologico Monzino, Functional Proteomics, Metabolomics, and Network Analysis, IRCCS, 20138 Milano, Italy; 2 Centro Cardiologico Monzino IRCCS, 20138 Milano, Italy; 3 Cardiovascular Section, Department of Clinical Sciences and Community Health, University of Milan, 20122 Milano, Italy; 4 Dipartimento di Biologia e Biotecnologie “Lazzaro Spallanzani”, Università di Pavia, 27100 Pavia, Italy

**Keywords:** Sacubitril/Valsartan, heart failure, miRNA

## Abstract

Sacubitril/Valsartan, used for the treatment of heart failure (HF), is a combination of two drugs, an angiotensin receptor inhibitor, and a neprilysin inhibitor, which activates vasoactive peptides. Even though its beneficial effects on cardiac functions have been demonstrated, the mechanisms underpinning these effects remain poorly understood. To achieve more mechanistic insights, we analyzed the profiles of circulating miRNAs in plasma from patients with stable HF with reduced ejection function (HFrEF) and treated with Sacubitril/Valsartan for six months. miRNAs are short (22–24 nt) non-coding RNAs, which are not only emerging as sensitive and stable biomarkers for various diseases but also participate in the regulation of several biological processes. We found that in patients with high levels of miRNAs, specifically miR-29b-3p, miR-221-3p, and miR-503-5p, Sacubitril/Valsartan significantly reduced their levels at follow-up. We also found a significant negative correlation of miR-29b-3p, miR-221-3p, and miR-503-5p with VO_2_ at peak exercise, whose levels decrease with HF severity. Furthermore, from a functional point of view, miR-29b-3p, miR-221-3p, and miR-503-5p all target Phosphoinositide-3-Kinase Regulatory Subunit 1, which encodes regulatory subunit 1 of phosphoinositide-3-kinase. Our findings support that an additional mechanism through which Sacubitril/Valsartan exerts its functions is the modulation of miRNAs with potentially relevant roles in HFrEF pathophysiology.

## 1. Introduction

The prevalence of heart failure (HF) with reduced ejection function (HFrEF) is continuously increasing in the general population, and still has a poor prognosis in the medium term. Indeed, in developed countries, notwithstanding the better management of cardiovascular disease, the overall incidence is increasing [1].

In patients with HFrEF, pharmacotherapy involves modulating renin-angiotensin-aldosterone (RAAS) and sympathetic nervous systems with angiotensin-converting enzyme inhibitors (ACE-I), beta-blockers, and mineralocorticoid receptor antagonists (MRA), which have beneficial effects on survival, hospitalization risk, and symptoms [1]. Recently, a few new drugs have been introduced in HFrEF therapy, showing an additional prognostic benefit on top of standard medical treatment. Among those, Sacubitril/Valsartan combines an angiotensin receptor blocker with a neprilysin inhibitor (angiotensin receptor–neprilysin inhibitors, ARNIs). In spite of its favorable effects on cardiac remodeling, functional capacity, and natriuretic peptides, its mechanisms of action are unclear [2,3]. Indeed, it has been suggested both hemodynamic and non-hemodynamic mechanisms may contribute to these effects. In this regard, we recently reported that Sacubitril/Valsartan affects lung diffusion capacity for carbon monoxide (DLCO) and lung mechanics; affects the release of the immature form of surfactant protein type B (proSP-B) from the alveolar-capillary membrane; and reduces the HF biomarkers, amino terminal pro-B-type natriuretic peptide (NT-proBNP) and soluble interleukin 1 receptor-like 1 (ST-2), suggesting the combined presence of hemodynamic and pleiotropic effects of the drug [4]. It is of note that proSP-B has emerged as a novel potential biomarker not only of alveolar-capillary membrane function but also of the overall HFrEF status [5,6,7]. Indeed, NT-proBNP, DLCO, and proSP-B circulating levels, which ameliorate with Sacubitril/Valsartan, all have a potential prognostic role in chronic HF [8], and are modulated by specific HFrEF treatments, such as levosimendan [9]. Furthermore, NT-proBNP reflects the hemodynamic status of the patients, while ST-2 mirrors the inflammatory and pro-fibrotic status of the disease [4,10].

To achieve a more mechanistic insight into the effects exerted by the therapy we analyzed the profiles of circulating microRNAs (miRNAs) in plasma from patients with HFrEF treated with Sacubitril/Valsartan for six months. miRNAs are short (22–24 nt) non-coding RNAs that pair accurately with their targets and promote the endonucleolytic cleavage of a single specific mRNA [11]. Circulating miRNAs, which are present in almost all biological fluids associated with microvesicles, high-density lipoproteins (HDL), or as parts of apoptotic bodies, are emerging as sensitive and stable biomarkers for various diseases, including cardiovascular diseases, neurodegenerative pathologies, and cancer [12]. Like intercellular miRNAs, circulating miRNAs participate in the regulations of several biological processes. Although the biological functions of extracellular miRNAs remain unclear, strong evidence suggests they are more than cellular waste products. Extracellular miRNA species may be involved in cell–cell signaling during various physiological and pathological processes [13]. Furthermore, miRNAs are showing promise as a diagnostic tool for a wide range of diseases prior to symptoms arising, and as a way to assess the response of a patient to therapy to aid in correcting and personalizing treatment [12].

## 2. Materials and Methods

### 2.1. Study Design

HFrEF outpatients referred to the Heart Failure Unit of Centro Cardiologico Monzino (CCM), eligible to start Sacubitril/Valsartan according to 2016 ESC Guidelines [14], were prospectively enrolled between December 2018 and December 2019. The present research protocol complied with World Medical Association Declaration of Helsinki, and it was approved by the Centro Cardiologico Monzino Ethical Committee (CCM 898). Each subject provided written consent to the study. Inclusion criteria were as follows: aged 18–80 years, males and females, New York Heart Association Class (NYHA) II-III in stable clinical condition, and left ventricular ejection fraction (LVEF) of ≤35%. Severe chronic obstructive pulmonary disease or the need for oxygen supplementation were considered exclusion criteria. Each patient underwent all study procedures at baseline while taking guideline-directed therapy for HF. After 36 h from the interruption of angiotensin-converting enzyme inhibitors or angiotensin receptor blockers, they started Sacubitril/Valsartan therapy with a 24/26 mg b.i.d. as the starting dose for all patients, progressively up titrated to 97/103 mg b.i.d. or the maximum tolerated dose, in a standard monthly-based fashion. All study procedures were also performed 6 months after the maximum tolerated dose was reached (T1). Specifically, patients underwent clinical assessment, lung function tests (standard spirometry and DLCO), venous blood sample collection, transthoracic echocardiography, a cardiopulmonary exercise test (CPET), and nocturnal cardiorespiratory monitoring, as previously described [4], and reported in Figure S1. Additional plasma samples from healthy subjects (HC) were selected from a cohort enrolled at Centro Cardiologico Monzino (CCM470). Each subject provided written consent to the study.

### 2.2. Plasma Preparation

Blood was drawn into Vacutainer tubes containing citrate 0.129 mol/L as an anticoagulant and was immediately prepared via centrifugation at 1500× *g* for 15 min at 4 °C. Plasma samples were then stored at −80 °C until use.

### 2.3. Total RNA Purification

Total RNA was extracted from plasma using the Total Rna Purification Plus Kit (Norgen, Thorold, ON, Canada), according to the manufacturer’s instructions.

### 2.4. MicroRNA Screening

Microarray analysis was performed by ThermoFisher with the GeneChip miRNA 4.0 Array (Life Technologies, Carlsbad, CA, USA), which contains 2578 human mature miRNA probe sets annotated in the miRBase 20 database. Data were analyzed using ExpressionSuite v1.1 software (Life Technologies, Carlsbad, CA, USA), using the global normalization method and considering as not expressed all miRNAs presenting Ct values of >35 in more than 50% of patients.

### 2.5. Single miRNA Assays

microRNA retro-transcription was conducted using a TaqMan Advanced miRNA cDNA Synthesis Kit (Life Technologies, Carlsbad, CA, USA), starting from 2 µL of total RNA extract. Selected miRNAs were evaluated using single TaqMan Advanced miRNA assays (Life Technologies, Carlsbad, CA, USA), following the manufacturer’s protocol. Data were calculated with the delta-delta Ct method, using miR-16-5p as the housekeeping gene.

### 2.6. Statistical Analysis

Statistical analysis was performed using SPSS 25.0 software (SPSS Inc., Chicago, IL, USA). Continuous variables were expressed as mean ± standard deviation (SD), mean ± standard error (SEM) where indicated, or median and [interquartile range] as appropriate, while discrete variables are shown as absolute numbers and percentages. Normality was evaluated using the Kolmogorov–Smirnov test and Shapiro–Wilk test. Comparisons between basal variables and end study variables were performed using paired t-tests for normally distributed variables, and the Wilcoxon signed rank test for non-normally distributed variables. All tests were 2-sided. A *p* of ≤0.05 was considered statistically significant.

Relationships between parameters of HF severity were evaluated using Spearman’s coefficient of rank correlation. Furthermore, relationships between HF severity variables and miRNA expression values adjusted for age were assessed via multivariable analysis (general linear model). An adjusted *p* of <0.05 was deemed statistically significant.

## 3. Results

### 3.1. Characteristics of the Study Population

The study was performed on a population of 69 patients enrolled to start Sacubitril/Valsartan therapy. The clinical variables have been previously described [4], and are briefly summarized in Table 1 and Table S1. Patients underwent all study procedures at baseline (T0), and 6 months (T1) after reaching the maximal dose. Total follow-up was 8.7 ± 1.4 months. For comparison, a small cohort of 10 healthy subjects (HC) was selected from a cohort enrolled at CCM (Table S2).

### 3.2. Profiling of Circulating miRNA Expression

To profile the circulating miRNAs differentially modulated by drug treatment, we screened 2578 miRNAs using TaqMan microRNA arrays in plasma samples from five HC and five HF patients (NYHA class II-III) at baseline and follow-up, selected from the global population based on the observed reduction in NT-proBNP and proSP-B (−75.1% ± 19.6% and −26.6% ± 10.1% reduction after treatment, respectively).

This screening revealed 443 detectable miRNAs in all conditions, among which only 5 were found to be putatively modulated by the disease and 10 modulated by treatment with Sacubitril/Valsartan (Table S3). According to the results of the screening, the availability of primers, and their detectability, we selected a total of five miRNAs, with two modulated by treatment (miR181a-1, and miR28-3p), two altered in HF patients with respect to control samples (miR320e, and miR503-5p), and miR450a-5p, which is modulated neither by the treatment nor by the disease.

### 3.3. Analysis of Selected Circulating miRNAs

We then validated them in the entire population, together with two miRNAs identified as potential HF biomarkers in a previous paper by D’Alessandra et al. [15] (miR221-3p and miR423-5p), and miRNA 29b-3p, described in the literature as a promoter of the pathologic hypertrophy of cardiac myocytes and overall cardiac dysfunction in HF [16].

Validation was performed via single plex assays on 10 HC and 69 HF subjects at baseline and after 6 months from the maximal dose. Results showed that only miR-29b-3p was upregulated in HF patients with respect to HC, and none of the validated miRNAs was modulated by Sacubitril/Valsartan treatment, when considering the entire population (Table 2).

**Figure 1:**
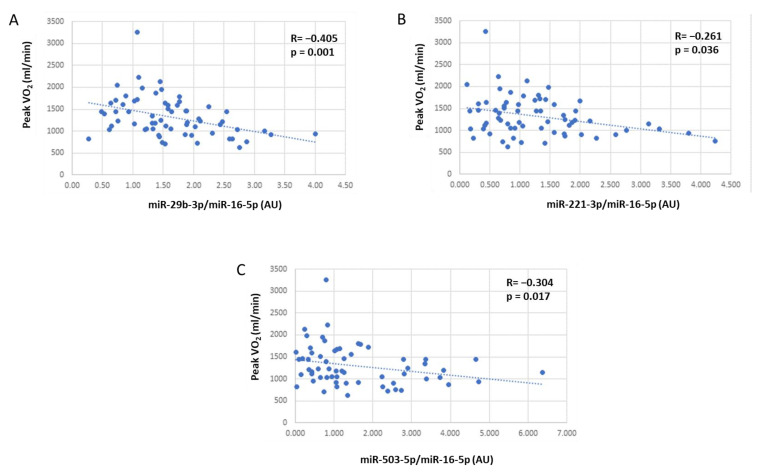
Correlation of (**A**) miR-29b-3p, (**B**) miR-221-3p, and (**C**) miR-503-5p with peak VO_2_ at baseline (n = 69 HF patients). miRNA levels are expressed as arbitrary units (AU) calculated with the delta-delta Ct method, using miR-16-5p as a housekeeping gene.

As we observed a broad distribution of the plasma levels of these miRNAs, we tested the association between them and the functional parameters normally evaluated in HF to follow the progression of the disease. As reported in Table 3, we observed a significant negative correlation of miR-29b-3p, miR-221-3p, and miR-503-5p with oxygen uptake at CPET (peak VO_2_), whose level decreases with HF severity (Figure 1). However, since miR-29b-3p and miR-221-3p were significantly correlated with age, we performed a multivariable analysis and we found that adjusting for age, both miRNAs are still significantly associated with peak VO_2_ (*p* = 0.004 and *p* = 0.024 for miR29 and miR221, respectively).

**Figure 2:**
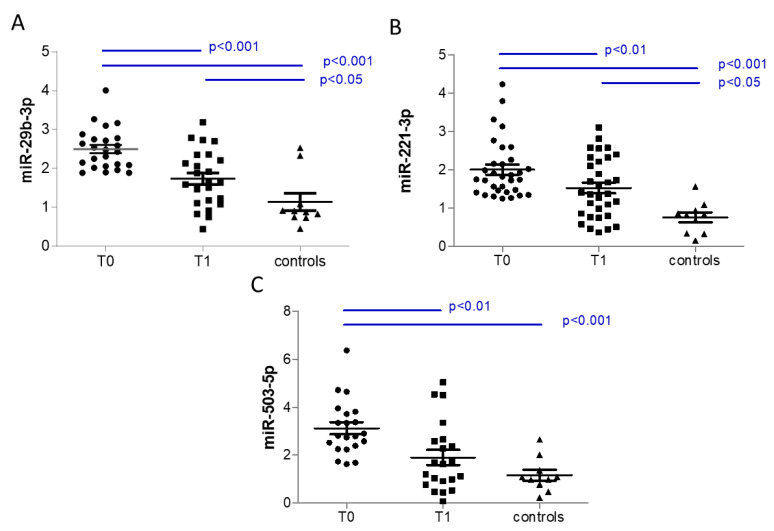
Effects of Sacubitril/Valsartan treatment on patients in the upper tertile of (**A**) miR-29b-3p (n = 24), (**B**) miR-221-3p (n = 32), and (**C**) miR-503-5p (n = 21) at baseline (T0) and after 6 months after the maximum tolerated dose was reached (T1). Data are expressed as mean ± SEM. *p*-value from paired t-test for comparison of T0 and T1. *p*-value from ANOVA for comparisons with control subjects (controls n = 10).

Considering all patients, we did not observe any correlation between the variation in validated circulating miRNAs levels, expressed as delta between follow-up and basal levels, and the variation in functional parameters expressed as delta between follow-up and basal levels (Table S4). However, considering the negative correlation with peak VO_2_, we focused on patients in the upper tertile for miR-29b-3p, miR-221-3p, or miR-503-5p, and we demonstrated that they have higher basal levels of miRNA than HC, and that these miRNAs are significantly reduced by Sacubitril/Valsartan at follow-up (Figure 2). It is of note that patients in the upper tertile for miR-29b-3p have significantly lower levels of peak VO_2_ in respect to those in the lower tertile at baseline (*p* < 0.001, 1450 [1142–1755] and 998 [826–1207], median [interquartile range] for lower and upper tertile, respectively) and at follow-up (*p* = 0.01, 1421 [1276–1904] and 1169 [947–1315], median [interquartile range] for lower and upper tertile, respectively). Similarly, patients in the upper tertile for miR-503-5p have significantly lower levels of peak VO_2_ in respect to those in the lower tertile at baseline (*p* = 0.011, 1448 [1109–11635] and 1056 [889–1393], median [interquartile range] for lower and upper tertile, respectively) and at follow-up (*p* = 0.011, 1386 [1212–1629] and 1200 [958–1335], median [interquartile range] for lower and upper tertile, respectively). In addition, the levels of peak VO_2_ for patients in the upper tertile for miR-221-3p tended to be higher than in patients in the lower tertile, even if not statistically significant neither at baseline (*p* = 0.190, 1454 [1036–1739] and 1188 [928–1482], median [interquartile range] for lower and upper tertile, respectively) nor at follow-up (*p* = 0.055, 1386 [1257–1880] and 1263 [989–1495], median [interquartile range] for lower and upper tertile, respectively).

Furthermore, the correlation between follow-up levels of functional or biochemical parameters and the validated miRNAs measured at follow-up revealed a positive correlation of interleukin ST-2 with miR-29b-3p, miR221-3p, miR320e, miR423-5p, and miR-450a-5p (Table 4).

### 3.4. Computational Analysis of miRNA Targets

**Figure 3:**
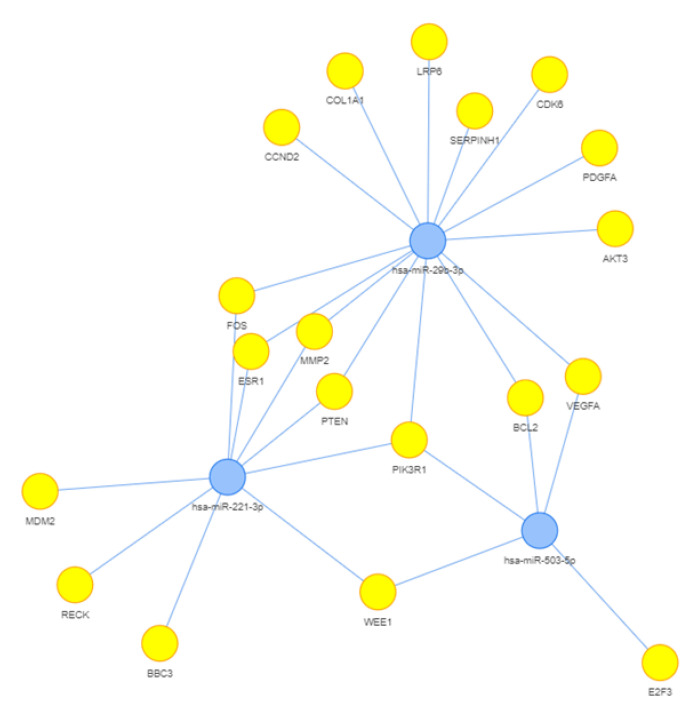
Network of miRNA targets.

To identify the potential functions of the three validated miRNAs that correlate with functional parameters (miR-29b-3p, miR-221-3p and miR-503-5p), we performed a targeted search using the Mienturnet tool and MiRTarBase “URL 27 March 2023, http://userver.bio.uniroma1.it/apps/mienturnet/”, searching for strong evidence of interaction between miRNA and target genes. As shown in Figure 3 reporting the network generated by the targets and the miRNAs, the three miRNAs share a common target, Phosphoinositide-3-Kinase Regulatory Subunit 1 (PIK3R1), and seven target genes are modulated by two miRNAs (Table S5).

**Figure 4:**
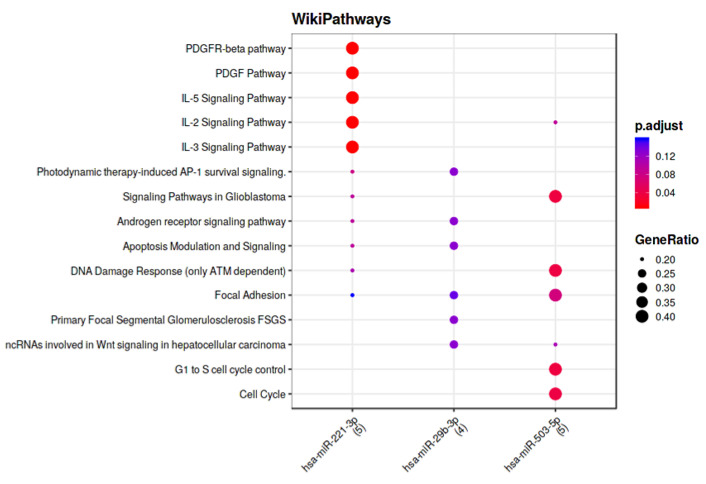
Functional enrichment of miRNA targets.

Furthermore, the functional enrichment analysis of the identified miRNA targets, performed using WikiPathways, revealed that the three miRNAs may have an impact on focal adhesion (Figure 4).

## 4. Discussion

In the present study, we assessed the effect of Sacubitril/Valsartan on circulating miRNAs in patients with stable HF. The main finding is that in patients with high baseline levels of miRNAs, specifically miR-29b-3p, miR-221-3p, and miR-503-5p, Sacubitril/Valsartan significantly reduced their levels at follow-up. We also found a significant negative correlation of miR-29b-3p, miR-221-3p, and miR-503-5p with peak VO_2_, whose levels are known to decrease in parallel with HF severity according to a functional impairment of the disease. These findings suggest that Sacubitril/Valsartan can affect miRNA levels in the most severe HFrEF. Furthermore, from a functional point of view, circulating miR-29b-3p, miR-221-3p, and miR-503-5p all target PIK3R1, which encodes regulatory subunit 1 of phosphoinositide-3-kinase [17].

The first study regarding the modulation of miRNAs by Sacubitril/Valsartan was performed in vitro in the plasma of rats [18]. The authors found that the treatment of cardiomyocytes, derived from the differentiation of induced pluripotent stem cells, with Sacubitril/Valsartan and Valsartan alone, increased the release of exosomes, which led to the downregulation of miR-181a. Furthermore, in vivo studies employing a rodent model of chronic myocardial injury demonstrated that miR-181a antagomir has a beneficial effect on cardiac functions by reducing myocardial fibrosis and hypertrophy and restoring the injured heart after myocardial infarction (MI) [18].

Recently, treatment with ARNI has been shown to reduce HF hospitalization and death in cardiac resynchronization therapy with defibrillator non-responder patients, through an epigenetic mechanism involving selected miRNAs (miR-181, miR-144, and miR-18) implicated in metabolic pathways of cardiac dysfunction [19].

Additionally, biochemical, molecular, and histopathological data revealed that Sacubitril/Valsartan exhibited protective effects against cyclophosphamide-induced oxidative and inflammatory events, which was most likely due to the changes in the levels of miRNA 150-3P and brain natriuretic peptide (BNP) [20].

Concerning the miRNAs modulated by Sacubitril/Valsartan, several findings support their roles in the pathophysiological mechanisms underlying HF. Indeed, a number of physiological and pathological processes are influenced by miR-29, including myogenesis, cardiac fibrosis, and tumorigenesis [21,22,23]. MiR-29 is also essential for the pathological remodeling of the cardiac extracellular matrix [24]. Additionally, several studies have shown different expression of miRNAs-29 in HF patients [25], likely modulating multiple genes involved in cell migration, invasion, apoptosis, and proliferation.

Recently, miR-29b-3p was found to inhibit cardiomyocyte proliferation in vitro and in vivo through the direct targeting of NOTCH2, a key regulator of cardiac development [26]. As a matter of fact, the evolutionarily conserved NOTCH signaling leads to heart malformations, including bicuspid aortic valve disease, calcification of the heart valves, Alagille syndrome, and ventricular septal defects [27,28].

The finding that the aberrant expression of miR-29b-3p could influence cardiac development via NOTCH2 highlights the role of epigenetic factors in the development of heart disease.

In previous studies, miR-221-3p has been implicated as a therapeutic target in HF by modulating the p27/CDK2/mTOR axis and cardiac remodeling [29]. Indeed, miR-221-3p inhibition promotes cardiac angiogenesis, alleviates myocardial hypertrophy, and further improves cardiac function in transverse aortic constriction-induced HF mice [30].

A significant upregulation of miR-221 is observed in patients with hypertrophic cardiomyopathy (HCM), and miR-221-3p expression in serum is negatively correlated with heart function in patients with HF [30]. The in vitro overexpression of miR-221 alone is sufficient to increase the size of cardiomyocytes, and the expression levels of the atrial natriuretic polypeptide (ANP) and BNP [31].

MicroRNA-503 (miR-503), which belongs to the miR-16 family [32], modulates various biological processes, and the dysregulation of miR-503 is associated with human diseases, especially cardiovascular disease, and cancer. Studies have shown that miR-503 is expressed abnormally in diabetes [33], pulmonary arterial hypertension, coronary artery disease [34], ischemic stroke [35], and cancer. Our results confirm previous observations performed by Lee Lee Wong, et al. (Abstract, “URL accessed on 27 March 2023 https://doi.org/10.1161/circ.132.suppl_3.15090”), who analyzed plasma miRNAs levels in 338 well-characterized HF patients and 208 age-matched controls without HF, and in a rat post-MI model. The authors found that among the 24 miRNAs highly up- or downregulated in clinical data, the levels of miR-503 were higher when comparing HF patients with non-HF controls (fold change = 1.69, *p* < 0.001). In the rat experimental model, levels of miR-503 (fold change = 2.21, *p* = 0.017) increased after post-MI on Day 2 and Day 7, but were not significant on Day 14 compared with a sham, thus indicating that circulating miR-503 is associated with HF. Nonetheless, these findings need further validation in studies with a larger number of subjects. Several studies reported that miR-503 was implicated in fibrosis (reviewed in [36]). Furthermore, in mouse cardiac fibrosis induced by transverse aortic constriction (TAC), and in mouse neonatal cardiac fibroblasts (CFs) treated with Angiotensin II [37], miR-503 expression was upregulated, and accompanied by cell proliferation and collagen production [37]. Conversely, treatment with antagomiR-503 in TAC mice improved cardiac function and decreased both transforming growth factor (TGF)-β and connective tissue growth factor (CTGF) expression, indicating that miR-503 has the potential to promote cardiac fibrosis [37].

Our results also showed a positive correlation, at follow-up, of interleukin ST-2 with miR-29b-3p, miR221-3p, miR320e, miR423-5p, and miR-450a-5p, which deserves some comments. ST2 is an interleukin (IL)-1 receptor family member with membrane-bound (ST2L) and soluble (sST2) isoforms, the latter being the form measured in our study. IL-33, an IL-1-related protein, once bound to ST2L, activates a signaling cascade that protects the myocardium against hypertrophy and cardiac fibrosis induced by pressure overload [38,39]. Conversely, sST2 acts as a decoy receptor for IL-33, preventing the IL-33/ST2L interaction, and the subsequent cardioprotective cascade of events. Furthermore, Van Vark et al. found that the repeated measurement of sST2 is a strong predictor of outcome in patients with acute heart failure, independently of NT-proBNP [40].

The positive correlation between ST-2 and miRNAs is in support of recent findings showing that much of the variation in sST2 production is driven by genetic factors. Indeed, Wang et al. reported that miR-487b reduces apoptosis, inflammatory responses, and fibrosis in HF by suppressing IL-33 through the inhibition of the IL-33/ST2 signaling pathway [41]. In the Bio-SHiFT (The Role of Biomarkers and Echocardiography in Prediction of Prognosis of Chronic Heart Failure Patients) study of 263 patients with chronic HF, repeatedly measured miR-22-3p contained important prognostic information and remained statistically significant after adjustment for temporal patterns of NT-proBNP, Troponin T, and CRP [42]. Although miR-22 has not directly been associated with the ST2/ IL-33 pathway, it nonetheless plays a critical role in the regulation of cellular proliferation, differentiation, and stress-induced hypertrophy [43]. Thus, as suggested by Patanè, combining miRNAs that influence the ST2/IL-33 pathway and repeatedly measuring ST2 has the potential to clarify the role of ST2 in patients with HF [44].

The potential mechanism of miR-503 in regulating cardiovascular disease involves the following targets: fibroblast growth factor (FGF)2, fibroblast growth factor receptor (FGFR)1, vascular endothelial growth factor (VEGF)A, TGF-β, CTGF, nuclear factor erythroid 2–related factor 2 (Nrf2), and phosphatidylinositol 3-Kinase (PI3K)/protein kinase B (Akt) in the pathological processes of cardiovascular diseases. It is also demonstrated that overexpression of miR-503 increased cell apoptosis and reactive oxygen species (ROS) production [35].

As anticipated, miR-29b-3p, miR-221-3p, and miR-503-5p all target PIK3R1, known as p85*α*, a regulatory subunit of class I PI3Ks [45]. It is of note that PI3Ks play a role in a variety of cardiovascular diseases and physiology, such as atherosclerosis, hypertension, angiogenesis, heart disease, and MI. As a result, these enzymes might also prove valuable as therapeutic targets [46]. The role of miRNA in the regulation of PI3KR1 was also described in the study by Zhan H. et al. [47], which showed that the downregulation of miR-128 restored PIK3R1 mRNA and the protein levels induced by angiotensin II, being accompanied by upregulation of the levels of phospho-Akt and -mTORC1 in vivo and in vitro. Moreover, alteration of the PIK3R1/Akt/mTORC1 pathway was negatively correlated with autophagy, while mTORC1 blocker rapamycin abolished miR-128 antagomir’s inhibition of angiotensin II-induced apoptosis and ROS production. Therefore, miR-128 downregulation alleviated the angiotensin-II-induced elevation of autophagy, likely via the PIK3R1/Akt/mTORC1 pathway in cardiomyocytes [47].

We recognize some limitations in this study. First, these data derive from subjects attending the local heart failure clinic and are therefore representative of a limited geographical area. Second, the cohort of subjects is small in size, indicating that the results should be addressed as preliminary findings, and, therefore, deserve validation with a larger sample size. Second, we did not consider sex as a factor that could correlate with the frequency of different miRNA in circulation, as demonstrated in an analysis of platelet-derived mRNA and miRNA [48], due to the low number of females among HF patients. Moreover, due to the limited sample size, we cannot completely exclude the impact of different ongoing therapies. We are aware that, albeit offering first insights into potential post-transcriptional functionalities of miRNAs, each of the bioinformatic tools for the prediction of miRNA targets has serious limitations [49]. However, an advantage of this approach is to reduce potentially large input lists to likely core interactions of the putative biological network, mainly in complex diseases, such as heart failure, a multiorgan disease (myocardium, kidney, lungs, and vascular vessels), which results from maladaptive signaling within intertwined molecular pathways. Indeed, the exact sources and location of circulating miRNAs need to be better defined. It should be noted that the majority of miRNAs in peripheral blood will likely be derived primarily from well-vascularized tissues, e.g., lungs and kidneys, in addition to blood cells themselves (platelets are a major contributor to the circulating RNA pool) [50]. Therefore, in vitro experiments with selected cells (i.e., cardiac, endothelial, or circulating cells) would not reflect the complexity of the disease physiopathology. From a technological point of view, several methods have been developed to quantitatively assess the levels of circulating miRNAs, and the establishment of standard operating procedures will help to reduce experimental variability and inter-study variability [51]. In conclusion, we demonstrated that the modulation of miRNA expression may be another mechanism through which Sacubitril/Valsartan exerts its pleiotropic effects. We found indeed for the first time that Sacubitril/Valsartan impacts the circulating miRNAs, with potentially important roles in the cardiovascular system.

Finally, miRNA research is now ready to move from laboratories to clinical trials with the use of high-throughput technologies. As a result, miRNAs may be used in diagnosis, prognosis, and to predict diseases, as well as being potentially used as therapeutic targets.

## Figures and Tables

**Table 1:** Characteristics of the study population at baseline.

Characteristic	Values at Baseline
Age (y)	64.8 ± 9.4
Male (n, %)	59 (85.5%)
BMI (kg/m^2^)	26.9 ± 4.4
Heart rate (bpm)	67.6 ± 11.1
Hemoglobin (g/dl)	14.3 ± 1.6
**Risk factors**	
Hypertension (n, %)	39 (56.5%)
Stroke (n, %)	4 (5.8%)
Smoker (n, %)	9 (13%)
Diabetes (n, %)	12 (17.4%)
COPD (n, %)	7 (10.1%)
Atrial fibrillation (n, %)	18 (26.1%)
**Therapy**	
ACE-I (n, %)	52 (75.4%)
ARBs (n, %)	15 (21.7%)
Beta blockers (n, %)	68 (98.6 %)
MRA (n, %)	48 (69.6%)
Diuretic (n, %)	55 (79.7%)
Ivabradine (n, %)	9 (13%)

BMI, body mass index; bpm, beats per minute; COPD, chronic obstructive pulmonary disease; ACE-I, angiotensin-converting enzyme inhibitors; ARBs, angiotensin receptor blockers; MRA, mineralocorticoid receptor antagonists. Data are expressed as mean ± SD.

**Table 2:** Levels of validated miRNAs in control subjects and HF patients at baseline (T0) or at follow-up (T1).

	Controls	HF T0	HF T1
**miRNA**			
miR-29b-3p	0.895 [0.745–1.4]	1.530 [1.030–2.090] *	1.470 [0.92–1.87]
miR-28-3p	0.645 [0.258–1.118]	0.985 [0.458–1.758]	1.150 [0.552–2.198]
miR-181a-1	0.655 [0.36–0.853]	0.945 [0.550–2.158]	1.197 [0.60–1.87]
miR-221-3p	1.360 [0.664–2.320]	1.045 [0.647–1.744]	1.169 [0.650–1.816]
miR-320e	1.118 [0.8–1.711]	0.889 [0.541–1.640]	0.89 [0.567–1.796]
miR-423-5p	0.860 [0.533–1.168]	0.875 [0.538–1.625]	1.062 [0.635–1.866]
miR-450a-5p	0.775 [0.408–1.320]	1.017 [0.545–1.863]	1.082 [0.575–1.797]
miR-503-5p	1.035 [0.703–1.525]	1.073 [0.524–2.357]	1.132 [0.492–2.292]

* *p* < 0.001 vs. control subjects by non-parametric Mann–Whitney test.

**Table 3:** Spearman’s correlation between basal levels of circulating miRNAs and clinical parameters at baseline.

		Age (years)	EF (%)	Peak VO_2_ (mL/min)	Peak VO_2_ (mL/min/kg)	DLCO (mL/min/mmHg)	ST-2 (ng/mL)	NT-proBNP (pg/mL)
**miRNA T0**								
miR-29b-3p	R	0.411 **	−0.132	−0.405 **	−0.371 **	−0.089	−0.067	0.119
	*p* values	0.001	0.279	0.001	0.003	0.477	0.673	0.332
miR-28-3p	R	0.277 *	0.043	−0.142	−0.178	−0.055	−0.086	−0.150
	*p* values	0.021	0.725	0.258	0.160	0.663	0.587	0.220
mir-181a-1	R	0.189	−0.012	−0.132	−0.170	−0.020	−0.125	−0.141
	*p* values	0.119	0.925	0.294	0.180	0.871	0.432	0.249
miR-221-3p	R	0.340 **	−0.026	−0.261 *	−0.271 *	−0.014	−0.052	−0.064
	*p* values	**0.004**	0.832	**0.036**	**0.030**	0.912	0.745	0.599
miR-320e	R	0.027	0.015	−0.068	−0.155	−0.060	−0.036	−0.138
	*p* values	0.823	0.906	0.592	0.221	0.632	0.819	0.260
miR-423-5p	R	0.155	−0.016	−0.123	−0.172	0.005	−0.116	−0.220
	*p* values	0.204	0.895	0.327	0.173	0.970	0.466	0.069
miR-450a-5p	R	−0.036	−0.083	−0.097	0.063	0.030	0.078	0.108
	*p* values	0.771	0.499	0.446	0.621	0.813	0.623	0.379
miR-503-5p	R	0.148	−0.061	−0.304 *	−0.209	−0.148	0.286	0.165
	*p* values	0.238	0.632	**0.017**	0.110	0.250	0.074	0.188

EF, ejection fraction; peak VO_2_, peak oxygen intake; VE/VCO_2_ slope, minute ventilation/carbon dioxide production relationship; DLCO, carbon monoxide lung diffusing capacity corrected for hemoglobin; ST-2, soluble interleukin 1 receptor-like 1; NT-proBNP, amino terminal pro-B-type natriuretic peptide. * *p* < 0.05; ** *p* < 0.01.

**Table 4:** Spearman’s correlation between levels of circulating miRNAs and clinical parameters at the follow-up.

		EF (%)	Peak VO_2_ (mL/min)	Peak VO_2_ (mL/min/kg)	DLCO (mL/min/ mmHg)	ST-2 (ng/mL)	NT-proBNP (pg/mL)
miRNA follow-up							
miR-29b-3p	R	0.003	−0.134	0.030	−0.166	0.357 **	0.141
	*p* values	0.982	0.291	0.816	0.196	**0.009**	0.252
miR-28-3p	R	0.184	−0.092	0.062	−0.094	0.189	0.000
	*p* values	0.132	0.469	0.625	0.465	0.176	0.998
mir-181a-1	R	0.049	−0.032	0.111	0.016	0.212	−0.029
	*p* values	0.690	0.802	0.381	0.904	0.127	0.815
miR-221-3p	R	0.051	−0.212	−0.045	−0.097	0.329 *	0.093
	*p* values	0.680	0.092	0.727	0.452	**0.016**	0.449
miR-320e	R	−0.160	−0.133	−0.072	−0.010	0.416 **	0.263 *
	*p* values	0.191	0.295	0.572	0.936	**0.002**	0.031
miR-423-5p	R	0.010	−0.052	−0.005	0.081	0.285 *	0.033
	*p* values	0.933	0.683	0.966	0.531	**0.039**	0.786
miR-450a-5p	R	−0.117	−0.011	0.154	0.067	0.419 **	0.058
	*p* values	0.343	0.932	0.225	0.608	**0.002**	0.641
miR-503-5p	R	0.189	−0.129	−0.006	−0.070	0.235	0.051
	*p* values	0.135	0.328	0.961	0.603	0.100	0.690

EF, ejection fraction; peak VO_2_, peak oxygen intake; VE/VCO_2_ slope, minute ventilation/carbon dioxide production relationship; DLCO, carbon monoxide lung diffusing capacity corrected for hemoglobin; ST-2, soluble interleukin 1 receptor-like 1; NT-proBNP, amino terminal pro-B-type natriuretic peptide. * *p* < 0.05; ** *p* < 0.01.

## Data Availability

Data collected in the study will be made available using the data repository Zenodo (“URL 1 April 2023, https://zenodo.org”), with restricted access, upon request to direzione.scientifica@ccfm.it.

## Supplementary Materials

The following supporting information can be downloaded at: https://www.mdpi.com/article/10.3390/biomedicines11041037/s1. Figure S1: Workflow of the study; Table S1: Characteristics of the study population after 6 months of treatment at the maximum dose of Sacubritil/Valsartan; Table S2: Characteristics of the healthy subjects enrolled at CCM; Table S3: List of miRNA modulated by treatment or by disease obtained from the miRNA screening; Table S4: Spearman’s correlation between variation of miRNAs and clinical parameters between follow-up and baseline; Table S5: miRNA targets identified using Mienturnet Tool.
