# Supplementary material for: Impact of Sacubitril/Valsartan on Circulating microRNA in Patients with Heart Failure
Source: Biomedicines. 2023 Mar 28;11(4):1037. doi: 10.3390/biomedicines11041037 (PMC10136141; doi:10.3390/biomedicines11041037)
Supplement: Supplementary file 1 [file biomedicines-11-01037-s001.zip › biomedicines-2240978-supplementary.pdf]

Supplementary Materials

Impact of Sacubitril/Valsartan on Circulating MicroRNA in Patients with Heart Failure

Maura Brioschi, Yuri D'alessandra, Massimo Mapelli, Irene Mattavelli, Elisabetta Salvioni, Sonia Eligini, Alice Mallia, Veronica Ricci, Erica Gianazza, Stefania Ghilardi, Piergiuseppe Agostoni and Cristina Banfi

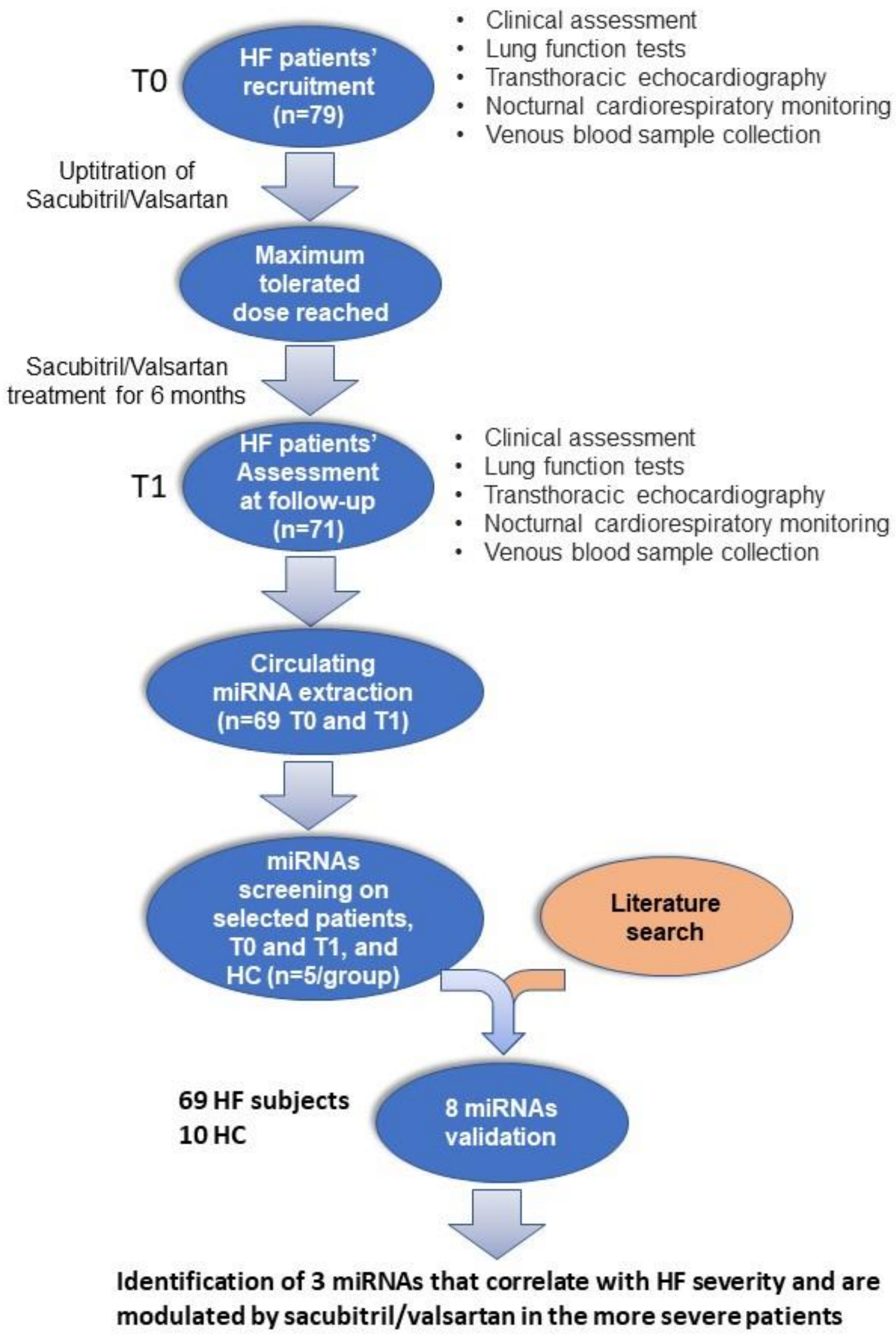

Figure S1. Workflow of the study. HF, heart failure; HC, healthy controls.

Table S1. Characteristics of the study population after 6 months of treatment at the maximum dose of Sacubitril/Valsartan.

Characteristic	baseline	Follow-up
Ejection fraction %	31.3 ± 5.2	36.9 ± 9.2
PAPs	34 ±12.3	28.2 ± 13.2
Hemoglobin (g/dl)	14.3± 1.6	14.13 ± 1.60
GFR (ml/min/1,73 m^2)	66.3 ± 16.5	64.6 ± 18.5
Sodium (mmol/L)	140.7 ± 2.6	140.97 ± 1.89
Potassium (mmol/L)	4.3 ± 0.4	4.35 ± 0.43
NT-proBNP (pg/mL)	1185 [663, 2878]	976 [401-1,634]*
ST-2 (ng/mL)	29.1 [18.8–37.1]	22 [15.2-31.9] *
proSP-B (AU)	57.9 [40.1 – 84.8]	50.4 [36.8 – 68.8]*
DLCO (ml/min/mmHg)	18.9 [15.7-23.6]	18.9 [15.5-24.4]
NYHA class I n	-	25
NYHA class II n	60	38
NYHA class III n	9	5

NYHA, New York Heart Association; PAPs, systolic pulmonary artery pressure; NT-proBNP, amino-terminal pro-B-type natriuretic peptide; GFR, glomerular filtration rate assessed by Modification of Diet in Renal Disease (MDRD) equation; Data are expressed as mean ± SD or median [interquartile range].

* $p<0.01$ by Wilcoxon rank test.

Table S2. Characteristics of the healthy subjects enrolled at Centro Cardiologico Monzino.

Characteristics	Values
Age (years)	52 ± 9
Males (n, %)	10 (100%)
BMI	25 ± 3.2
NT-proBNP (pg/ml)	20 [15–35]
Hemoglobin (g/dl)	14.55 ± 0.88
Creatinine (mg/dl)	0.96 ± 0.22
GFR (ml/min/1,73 m^2)	86.8 ± 19.5
Sodium (mmol/l)	141 ± 1.8
Potassium (mmol/l)	4.06 ± 0.35
**Therapy**	
ACE-I (n, %)	1 (10%)
ARBs (n, %)	2 (20%)
Statins (n, %)	1 (10%)
**Pulmonary function**	
FVC (L)	5.161 ± 0.64
FVC%	102.1 ± 8.5
FEV1 (L)	4.04 ± 0.52
FEV1%	102.4 ± 7.9
FEV1/FVC	78.6 ± 6.5
DLCO (mL/mm Hg/min)	32.9 ± 11.1
DLCO %	100.2 ± 25.4
DM (mL/mm Hg/min)	26.7 ± 16.02
VC (mL)	102 ± 29.7
VA (L)	6.9 ± 1.6

BMI, body mass index; NT-proBNP, amino terminal pro-B-type natriuretic peptide; GFR, glomerular filtration rate assessed by Modification of Diet in Renal Disease (MDRD) equation; ACE-I,

angiotensin-converting-enzyme inhibitors; ARBs, angiotensin receptor blockers; FEV_1, forced expiratory volume in 1 second; FVC, forced vital capacity (FVC); DLCO, carbon monoxide lung diffusing capacity; DM, membrane diffusion; VC, amount of blood participating in gas exchange; VA, alveolar volume. DLCO values were corrected for hemoglobin concentration and were expressed as a percentage of predicted values, as for FVC and FEV_1.

Table S3. List of miRNA modulated by treatment or by disease obtained from the miRNA screening.

ID	Fold Change	P-val	Transcript ID	Accession	Chromosome	Strand
**miRNA modulated in HF patients in response to drug treatment**						
20518802	1,9	0.00	hsa-miR-4430	MIMAT0018945	chr2	+
20519610	-1,78	0.01	hsa-miR-4750-3p	MIMAT0022979	chr19	+
20523001	-1,48	0.01	hsa-miR-6069	MIMAT0023694	chr22	-
20524036	1,8	0.02	hsa-miR-6126	MIMAT0024599	chr16	-
20525459	1,38	0.00	hsa-miR-6749-5p	MIMAT0027398	chr11	-
20525511	1,47	0.00	hsa-miR-6775-5p	MIMAT0027450	chr16	-
20525719	-1,21	0.01	hsa-miR-6879-5p	MIMAT0027658	chr11	+
20500159	1,36	0.00	hsa-miR-28-3p	MIMAT0004502	chr3	+
20534530	1,28	0.01	hsa-mir-181a-1	MI0000289	chr1	-
20535692	1,38	0.00	hsa-mir-941-1	MI0005763	chr20	+
**miRNA modulated in HF patients in respect to control healthy subjects**						
20523020	4.32	1.09E-05	hsa-miR-6088	MIMAT0023713	chr19	+
20503882	2.71	0.001	hsa-miR-503-5p	MIMAT0002874	chrX	-
20523019	1.98	0.0026	hsa-miR-6087	MIMAT0023712	chrX	+
20506787	4.01	7.53E-05	hsa-miR-1237-5p	MIMAT0022946	chr11	+
20536308	-1.56	0.024	hsa-mir-320e	MIMAT0015072	chr19	-

Table S4. Spearman correlation between variation of miRNAs and clinical parameters between follow-up and baseline.

		delta-EF	delta-VO_2 peak	delta-VO_2p / Kg	delta-VEVCO2 SLOPE	delta-DLCO / Hb	delta-ST2	delta-NTproBNP
**miR-28-3p-delta**	R	-0.004	0.061	0.151	0.152	-0.061	-0.239	0.008
	p value	0.976	0.630	0.238	0.231	0.646	0.128	0.946
**miR-29b-3p-delta**	R	-0.126	0.134	0.136	-0.064	0.126	-0.144	-0.110
	p value	0.307	0.293	0.288	0.616	0.342	0.365	0.370
**miR-181a-5p-delta**	R	0.008	0.131	0.217	0.172	-0.069	-0.209	-0.025
	p value	0.945	0.302	0.087	0.173	0.606	0.184	0.839
**miR-221-3p-delta**	R	0.038	0.170	0.161	0.029	0.029	-0.183	-0.035
	p value	0.761	0.180	0.207	0.823	0.829	0.247	0.776
**miR-320e-delta**	R	-0.067	0.109	0.186	,292*	-0.199	-0.040	0.047
	p value	0.587	0.393	0.145	0.019	0.131	0.801	0.703
**miR-423-5p-delta**	R	-0.035	0.129	0.207	0.228	-0.088	-0.043	-0.006
	p value	0.778	0.310	0.104	0.069	0.505	0.785	0.962
**miR-450a-5p-delta**	R	-0.047	**0.251***	**0.348****	0.187	-0.067	-0.061	0.102
	p value	0.708	**0.047**	**0.006**	0.143	0.620	0.703	0.412
**miR-503-5p-delta**	R	0.190	0.155	0.179	0.096	0.116	0.095	-0.047
	p value	0.133	0.236	0.175	0.467	0.401	0.558	0.715

Table S5. miRNA targets identifed by Mienturnet Tool.

microRNA	microRNA Target Gene
hsa-miR-221-3p	BBC3
hsa-miR-221-3p	FOS
hsa-miR-221-3p	ESR1
hsa-miR-221-3p	PTEN
hsa-miR-221-3p	WEE1
hsa-miR-221-3p	RECK
hsa-miR-221-3p	MDM2
hsa-miR-221-3p	PIK3R1
hsa-miR-221-3p	MMP2
hsa-miR-29b-3p	COL1A1

hsa-miR-29b-3p	CDK6
hsa-miR-29b-3p	BCL2
hsa-miR-29b-3p	VEGFA
hsa-miR-29b-3p	ESR1
hsa-miR-29b-3p	MMP2
hsa-miR-29b-3p	PTEN
hsa-miR-29b-3p	FOS
hsa-miR-29b-3p	PIK3R1
hsa-miR-29b-3p	CCND2
hsa-miR-29b-3p	SERPINH1
hsa-miR-29b-3p	PDGFA
hsa-miR-29b-3p	LRP6
hsa-miR-29b-3p	AKT3
hsa-miR-503-5p	WEE1
hsa-miR-503-5p	VEGFA
hsa-miR-503-5p	PIK3R1
hsa-miR-503-5p	BCL2
hsa-miR-503-5p	E2F3
